# Pedestrian navigation activity recognition method based on two-stream transformer and contrastive learning

**DOI:** 10.1016/j.isci.2026.115252

**Published:** 2026-04-01

**Authors:** Qu Wang, Junying Ma, Meixia Fu, Jianquan Wang, Yuntian Brian Bai, Zhuqing Jiang, Hongdun Li

**Affiliations:** 1 School of Automation Science and Electrical Engineering, University of Science and Technology Beijing, Beijing 100083, China; 2 Shunde Graduate School, University of Science and Technology Beijing, Foshan 528399, China; 3 School of Science, STEM College, RMIT University, Melbourne, VIC, Australia; 4 School of Artificial Intelligence, Beijing University of Posts and Telecommunications, Beijing 100876, China; 5 China Academy of Transportation Sciences, Beijing 100029, China

**Keywords:** computer science, engineering

## Abstract

Pedestrian navigation activity recognition (PNAR) serves a pivotal role in in the pedestrian positioning and navigation field, providing strong technical support for various aspects such as pedestrian dead reckoning, and multi-source information fusion positioning. This paper proposes a PNAR method that combines a two-stream convolutional transformer architecture with self-supervised contrastive pretraining to address challenges in learning robust, transferable, and generalizable representations from sensor data. The spatial stream captures multi-modal sensor dependencies, while the temporal stream leverages attention mechanism to excavate temporal relationships. The two-stream design effectively processes multi-modal sensor data and models complex activities. Contrastive pretraining leverages unlabeled data to learn invariant and transferable representations, significantly enhancing generalization across datasets. The proposed method was evaluated on four public datasets, achieving exceptional performance—99.08% accuracy and 99.22% F1-score, outperforming existing PNAR methods, including CNNLSTM + Attention and Transformer-based PNAR models. Furthermore, we conducted cross-dataset experiments on data with different sensor configurations and activity labels to validate the model’s superior generalization ability.

## Highlights

• TSCT model temporal and spatial dependencies of inertial sensor data
• We design a Self-supervised contrastive pretraining framework
• Conduct extensive cross-dataset experiments to validate generalization capabilities

## Graphical abstract

**Figure:**
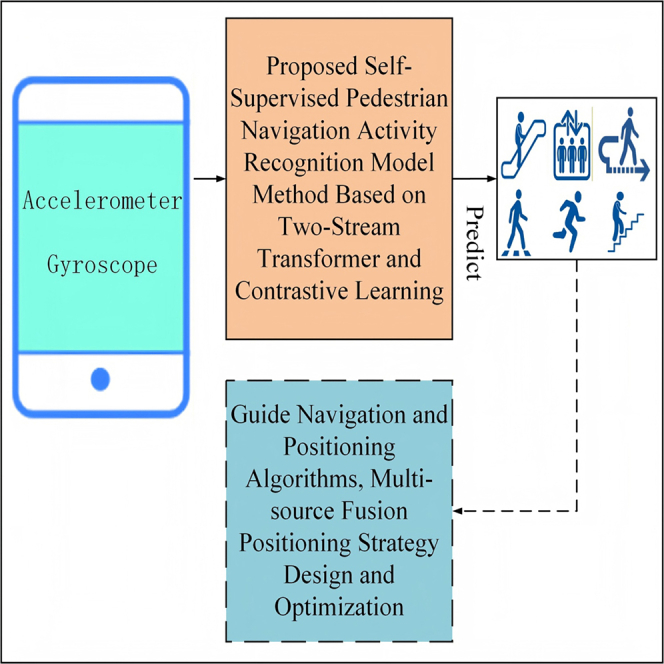

## Introduction

Human activity recognition (HAR) based on smartphone sensors has been an attractive research topic due to its extensive applications such as smart security,^1^ healthcare monitoring,^2^ smart homes,^3^ and human-computer interaction.^4^ Pedestrian navigation activity recognition (PNAR) is a subset of HAR.^5^ In the field of pedestrian positioning and navigation, Accurate recognition of pedestrian navigation activity (such as static, normal walking, running, jumping, upstairs, downstairs, and cycling) is the core technical foundation for achieving high-precision, scene-adaptive navigation and positioning^4^: (1) motion characteristics compensate for sensor errors. Pedestrian movement patterns such as walking, running, and going up/down stairs exhibit regularity (such as periodic step frequency and step length), which can be used to calibrate the cumulative errors of inertial measurement unit (IMU) through pattern recognition.^6^ (2) Motion state constrains intelligent fusion of multi-source information.^7^^,^^8^ In multi-source fusion positioning such as IMU/GNSS/Bluetooth/Wi-Fi/vision, motion patterns can serve as state observation measurements while assisting in selecting the optimal positioning source. (3) Enhanced robustness in complex environments. When GNSS signals are blocked in urban canyons, pedestrian dead reckoning mode combined with building layout constraints (such as sidewalk direction) can limit trajectory drift. In indoor scenarios, recognizing user activity patterns of taking elevators/escalators can prevent floor misjudgment (traditional altimeters are susceptible to air pressure fluctuations).^9^ (4) User intent understanding. Motion patterns can infer user behavioral intentions (such as “stopping to look around” possibly corresponding to interest points), providing contextual information for location-based services (LBSs). In mall navigation, recognizing “wandering” patterns can trigger promotional information. (5) Energy consumption and computational efficiency optimization. Outdoor activities (such as cycling) switch to GPS-dominated mode, reducing unnecessary computation; indoor activities switch to multi-source information fusion mode, improving positioning accuracy; when a stationary state is detected, high-energy modules such as GNSS are automatically turned off while reducing sampling frequency of simple sensors. Therefore, accurate and robust pedestrian navigation activities recognition (PNAR) help design and optimize positioning models to improve positioning accuracy while reducing energy consumption.

According to the different data acquisition devices, current PNAR methods fall into following three major groups: video image-based PNAR methods, radio frequency signal-based PNAR methods, and inertial sensor-based PNAR methods.^10^^,^^11^ The methods based on video images recognizes people’s activity through video and images collected by cameras. This way has the following own defects: one is that the light intensity has a greater impact on the camera. The same activity is different for videos or images under different lighting. At night, the camera cannot even capture any information; the second is that its recognition range is limited. Once the range of the camera is exceeded, the camera cannot collect video or images; the third is that the distance between the recognized person and the camera is different, which increases the difficulty of modeling.^12^ Radio frequency signal-based activity recognition is to obtain various data such as the azimuth, distance, speed, shape, and size of the target through the electromagnetic waves returned by the radar, thereby completing activity recognition.^13^ The disadvantages of this method: one is that the price of the radar is relatively expensive; the other is that it is easily affected by other targets and the environment. Inertial sensors can capture people’s motion state very well, without being limited by light, range, and environment. In addition to the rich built-in sensors and strong storage and computing capabilities, the smartphones carried by users can also avoid the disadvantage of being uncomfortable to wear and become the best carrier for ubiquitous activity recognition.^14^^,^^15^

This study focuses on PNAR using inertial sensors embedded in smartphones. Extensive research has been conducted on sensor-based PNAR using classical machine learning models, including decision trees (DTs), K-nearest neighbors (KNNs), hidden Markov models (HMMs), and support vector machines (SVMs). Feature extraction and selection constitute the core components of machine learning-based PNAR methodologies, as they directly influence the discriminative power and generalization capability of the subsequent classification tasks. Deep learning-driven end-to-end PNAR methods automatically extract effective features from raw data, significantly improving recognition accuracy. So far, research on activity recognition is no longer limited to a single network model architecture. It has developed from the simplest machine learning method^16^^,^^17^^,^^18^ to simple deep learning network,^19^^,^^20^^,^^21^^,^^22^ and then used hybrid deep learning networks.^23^^,^^24^^,^^25^ Recently, attention-based models have attracted considerable interest in PNAR tasks.^26^^,^^27^^,^^28^^,^^29^^,^^30^^,^^31^ Contrastive learning is a self-supervised learning method that helps models to understand sample similarities by comparing pairs of distinct samples. Contrastive learning has shown its powerful representation learning capabilities in many fields. Inspired by the strong advances achieved by contrastive self-supervised learning methods in computer vision (MoCo,^32^ SimCLR,^33^ SimSiam,^34^ and BYOL^35^), researchers have increasingly adapted these techniques to sensor-based PNAR.^36^^,^^37^^,^^38^

Despite the extensive research carried out on activity recognition using smartphone-built inertial sensors, several challenges and difficulties remain unresolved: (1) different human have obvious differences in height, weight, walking habits, etc. The built-in sensors in terminals have many types, varying accuracy, and lack of necessary calibration. Existing activity recognition models struggle to adapt to heterogeneous users and devices^39^; (2) recognition accuracy is closely related to the device’s posture. There are differences in data distribution between sensor data collected under different location conditions, resulting in the performance of the trained activity recognition model being unable to be guaranteed when facing sensor data collected at other locations.^40^^,^^41^ However, collecting a large amount of labeled data for each location and retraining an activity recognition model will consume a lot of resources and is also unrealistic.^42^

This paper presents a novel PNAR method by introducing a two-stream convolutional transformer (TSCT) architecture combined with self-supervised contrastive pretraining, addressing key challenges in learning robust, transferable, and generalizable representations from sensor data. The dual-stream design captures spatial dependencies through multi-modal sensor integration and temporal relationships using self-attention mechanisms, enabling effective modeling of complex activity. The contrastive pretraining method leverages unlabeled data, significantly reducing reliance on labeled datasets and improving cross-dataset generalization. The primary contributions of our research are listed below:

(1) We propose a novel architecture to simultaneously model temporal and spatial dependencies in inertial sensor data. The spatial stream captures multi-modal sensor dependencies, while the temporal stream employs an attention mechanism to effectively excavate temporal relationships. This dual-stream design enables the model to comprehensively process complex activities, outperforming single-stream models in capturing diverse dependencies.
(2) We propose a self-supervised contrastive pretraining method based on the Bootstrap Your Own Latent (BYOL) framework to achieve robust PNAR using a small amount of labeled data. We employ a two-stream Transformer as the encoder for BYOL to learn high-level feature representations from unlabeled data during the self-supervised pre-training phase. Subsequently, we utilize a small amount of labeled data for supervised fine-tuning to train a simple linear classifier that classifies the features learned by the encoder, thereby achieving accurate recognition. This method demonstrably enhances model generalization capabilities when applied to cross-dataset scenarios featuring heterogeneous sensor modalities and divergent activity distributions.
(3) We conduct extensive cross-dataset experiments demonstrate the proposed method’s exceptional generalization capacity. Experimental results show consistently superior performance across four public datasets (WISDM, UCI-HAR, PAMAP2, and KU-HAR) with minimal reliance on labeled data. This robust cross-dataset generalization capability, coupled with minimal supervision requirements, underscores the method’s practical viability for real-world positioning and navigation systems.

## Results

We carried out comprehensive experiments on public datasets to assess how well the proposed method works. Four widely used public datasets are first described. We detail model parameters. We provide a thorough evaluation of the proposed method’s performance. We trained proposed method on one dataset and tested on another to validate its generalization ability. We evaluate the performance of the proposed method under different scales of labeled data. We reproduced eight baseline models, and conducted comparative experiments.

### Datasets for benchmarking

**Figure 1:**
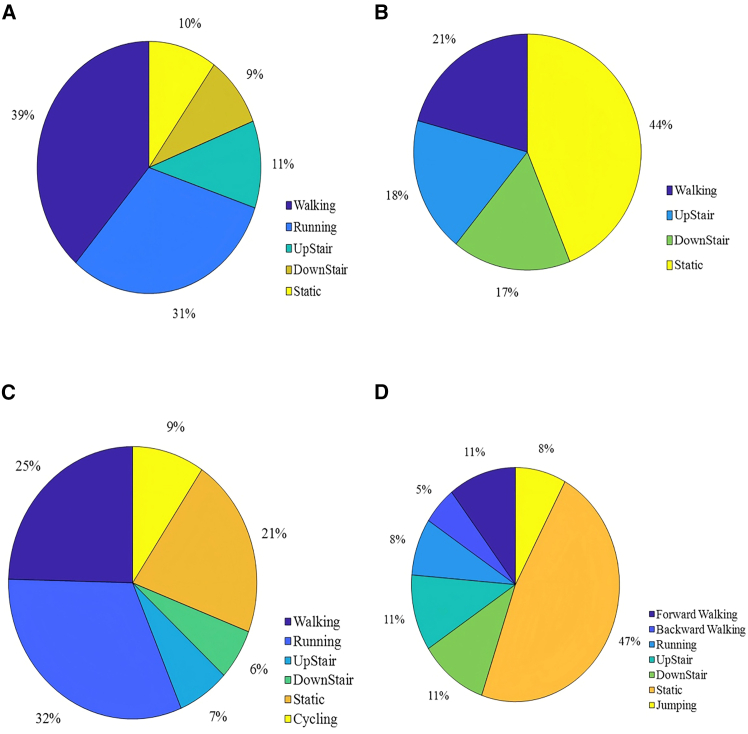
Distribution of proportions of different activities (A) WISDM. (B) UCI-HAR. (C) PAMAP2. (D) KU-HAR.

To assess the proposed method’s performance, which uses TSCT and contrastive learning, we conducted experiments on several widely used public datasets. These datasets exhibit significant differences in sensor configuration, activity categories, and physiological characteristics of subjects, which can effectively validate model’s robustness and generalization ability. Table 1 briefly introduces four public datasets, where Acc, Gyr, and Mag represent the accelerometer, gyroscope, and magnetometer, respectively. Figure 1 presents the proportion of different activities in each dataset. The detailed introduction of each dataset is as follows:

**Table 1:** Summary of dataset

DataSets	Frequency	Users	Selected activities	Devices	Sensors	Position	Samples
WISDM^43^	20 Hz	36	5 (walking, jogging, upstairs, downstairs, static)	iPhone	Acc	Front pocket of pants	1098207
UCI-HAR^44^	50 Hz	30	4 (walking, upstairs, downstairs, static)	Galaxy S Ⅱ	Acc, Gyr	Belt	815614
PAMAP2^45^	100 Hz	9	6 (static, walking, running, bicycling, upstairs, downstairs)	3 Colibri wireless inertial measurement units (IMUs)	Acc, Gyr, Mag	Right arm, chest, and ankle	2844868
KU-HAR^46^	100 Hz	90	7 (static, forward walking, backward walking, running, jumping, upstairs, downstairs)	Samsung Galaxy J7, Redmi note4, Realme 3 Pro, Realme 5i, Realme C3	Acc, Gyr	Belt	6306335

WISDM^43^: this dataset contains data from smartphones carried in users’ pockets during activities like walking, jogging, and stair climbing. The low sampling frequency (20 Hz) and unconstrained device placement make it a challenging dataset, useful for evaluating the model’s adaptability to real-world low-resolution data.

PAMAP2^45^: this dataset contains data from wearable devices positioned on different body parts, capturing a diverse range of 18 activities such as walking, running, cycling, and household chores. The high-frequency data (100 Hz) from accelerometers, gyroscopes, and magnetometers provides rich temporal and spatial features, making it an ideal dataset to evaluate both the spatial and temporal modeling capabilities of the proposed method.

UCI-HAR^44^: this dataset is widely used for HAR benchmarking, featuring six common activities (e.g., walking, sitting, and standing) recorded using accelerometers and gyroscopes embedded in smartphones. Its relatively small size and simple activity classes make it a suitable baseline for evaluating the core effectiveness of the method.

KU-HAR^46^: the dataset is a smartphone sensor-based HAR dataset released by Khulna University in 2020. This dataset records 18 activities from 90 participants. Among the 90 participants, 75 were male and 15 were female, with age spanning of 18–34 and an average age of 21.7.

These datasets collectively offer a comprehensive benchmarking environment to test the model’s effectiveness across a spectrum of PNAR challenges, including multimodal integration, temporal dynamics, inter-subject variability, and device heterogeneity.

### Model parameters

During the pre-training stage, the TSCT model serves as the encoder, where the deep convolutional blocks employ 64, 128, 256, and 512 filters, respectively, with a uniform kernel size of three. The convolutional feedforward module incorporates 512 filters with kernel sizes of three and one. BYOL is adopted as the contrastive learning framework; both the projector and predictor utilize MLP layers of dimension 512, and the exponential moving average coefficient is set to 0.991. Optimization is performed using Adam with an initial learning rate of 3 × 10^−4^, which is adaptively scheduled following a simulated cosine annealing strategy. Pre-training relies exclusively on unlabeled data, with each batch comprising 512 samples, and the training process is capped at 50 epochs.

In the subsequent fine-tuning phase, the pre-trained TSCT model is employed solely for feature extraction, upon which a linear classifier is appended. To assess the efficacy of the pre-training, linear evaluation is conducted by freezing all parameters of the TSCT encoder and training only the linear classification layer using a limited set of labeled samples. The initial learning rate is set to 3 × 10^−3^ and follows the same simulated cosine annealing adjustment policy.

The number of model parameters and the number of FLOPs is 73.22 M and 22.14 M, respectively. This is mainly reflected in model training, but offline training is often cost-insensitive. The online application costs of proposed method are acceptable and can be run on smartphones.

### Experiment results on public datasets

To avoid accidental results, we use five repetitive leave-one-subject-out cross-validation (LOSO-CV) tests to evaluate the performance of proposed method. Table 2 provides a comprehensive summary of the proposed method’s performance across four benchmark datasets. The proposed method consistently achieved high classification metrics, with accuracy ranging from 98.62% (KU-HAR) to 99.51% (UCI-HAR). Precision, recall, and F1 score metrics similarly indicate robust recognition capabilities, with all values exceeding 98% across datasets. Notably, the proposed method excelled in datasets with well-separated dynamic activities, such as WISDM (F1 score of 99.21%), while slightly lower performance was observed on datasets with complex activities like KU-HAR (F1 score of 98.26%). The proposed method has good learning ability on four different datasets, and the average recognition accuracy can reach 99.08%. The experimental findings verified the proposed method’s performance, demonstrating its robustness and reliability in PNAR tasks.

**Table 2:** Performance metrics on individual datasets

Dataset	Accuracy (%)	Precision (%)	Recall (%)	F1 score (%)
WISDM	99.32 ± 1.11	99.11 ± 1.18	99.14 ± 1.21	99.12 ± 1.03
UCI-HAR	99.51 ± 1.23	99.25 ± 1.14	99.17 ± 1.16	99.21 ± 1.08
PAMAP2	98.87 ± 1.27	98.93 ± 1.23	98.62 ± 1.21	98.77 ± 1.23
KU-HAR	98.62 ± 1.38	98.39 ± 1.25	98.14 ± 1.22	98.26 ± 1.29
Average	**99.08** ± 1.25	**98.92** ± 1.20	**98.77** ± 1.20	**99.22** ± 1.16

Bold shows the average performance on different datasets.

**Figure 2:**
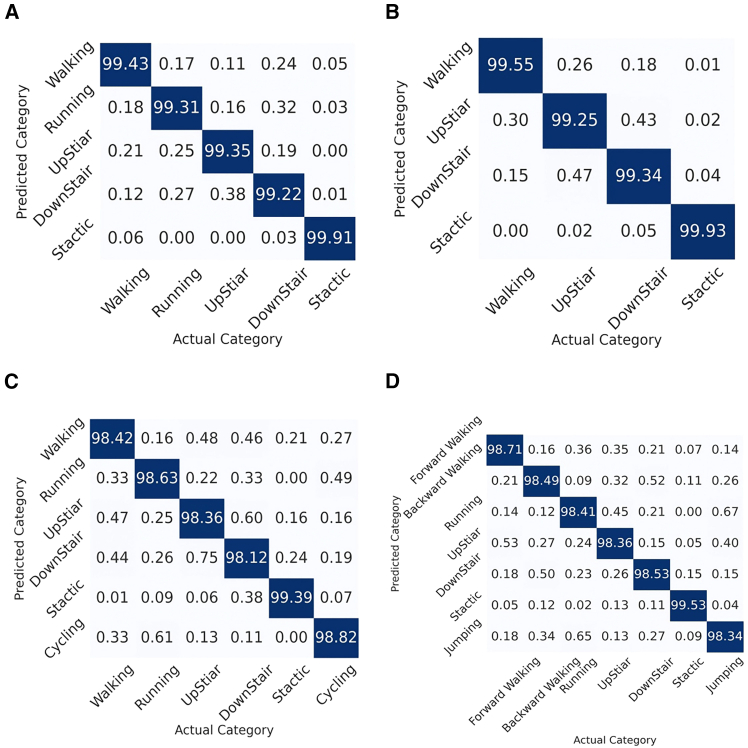
Different datasets’ confusion matrix (A) WISDM. (B) UCI-HAR. (C) PAMAP2. (D) KU-HAR.

Figure 2 illustrates the distribution of proportionate predictions across different activity categories for four distinct datasets. These confusion matrices visualize the predictive accuracy of each activity category by displaying the percentage of predictions (rows) that correctly match the actual categories (columns). From the confusion matrix, we can see that the values on the main diagonal are much larger than the rest of the values in the matrix, which shows that the proposed method has outstanding accuracy and effectively distinguish different activity categories.

### Cross-dataset generalization capability validation

We evaluate the cross-dataset generalization capability of the proposed method by training the proposed PNAR model on one dataset and testing it on another dataset. The results shown in Table 3, demonstrate the robustness of the proposed method in generalizing across datasets. When trained on the PAMAP2 and tested on WISDM, the proposed method achieved 97.67% accuracy, 96.88% precision, 97.65% recall, and 97.26% F1 scores. Similarly, when tested on UCI-HAR, the proposed method achieved slightly higher metrics, including 97.89% accuracy and 97.60% F1 score. Notably, when trained on KU-HAR, the proposed method exhibited superior generalization, achieving 98.17% accuracy on WISDM and 98.23% accuracy on UCI-HAR, with F1 scores consistently exceeding 97.30%, highlighting its adaptability to datasets with differing activity distributions and sensor setup. These results underscore the model’s ability to learn invariant and transferable features across datasets with varying complexities and sensor modalities, demonstrating its robustness for real-world activity recognition applications.

**Table 3:** Performance metrics on cross-dataset

Train dataset	Test Dataset	Accuracy (%)	Precision (%)	Recall (%)	F1 score (%)
PAMAP2	WISDM	97.67	96.88	97.65	97.26
	UCI-HAR	97.89	97.23	97.97	97.60
KU-HAR	WISDM	98.17	97.31	97.48	97.39
	UCI-HAR	98.23	97.95	97.69	97.82

### Robustness to annotation scarcity

To evaluate the performance of the proposed method under different scales of labeled data, the proposed model was trained using 2%–20% of labeled data, respectively. Table 4 summarizes the accuracy variations on the WISDM, UCI-HAR, PAMAP2, and KU-HAR datasets. As the amount of labeled data increases from 2% to 20%, the recognition accuracy shows a significant improvement. Due to the smaller sample size of the UCI-HAR dataset, the performance of the proposed method is inferior to the other three datasets when the labeling data ratio is low. The model trained with 12% labeled data achieves recognition accuracies of 99.32%, 99.51%, 98.87%, and 98.62% on the WISDM, UCI-HAR, PAMAP2, and KU-HAR datasets, respectively, indicating that the model has good fast convergence.

**Table 4:** Mean accuracy (%) of proposed methods trained by different labeled data on individual datasets

Dataset	2%	4%	6%	8%	10%	12%	14%	16%	18%	20%
WISDM	56.34	68.65	83.24	92.32	98.91	**99.32**	99.33	99.36	99.35	99.37
UCI-HAR	47.61	58.84	67.26	82.58	94.37	**99.51**	99.53	99.53	99.52	99.55
PAMAP2	57.43	71.87	89.12	95.45	98.37	**98.87**	98.91	98.94	98.97	99.03
KU-HAR	62.67	86.39	93.76	97.33	98.22	**98.62**	98.68	98.70	98.70	98.73
Average	56.01	71.44	83.35	91.92	97.47	**99.08**	99.12	99.13	99.13	99.17

Bold shows the impact of different training data volumes on performance. Training with 12% labeled data yields good performance, and further augmentation of the training data provides only limited performance improvement.

### Comparison with other methods

To assess the advanced performance, we reproduced eight baseline models, which included conventional machine learning methods like KNN and SVM, along with deep learning methods like CNN, LSTM, and transformer. Comparative experiments were performed based on the datasets mentioned earlier. Table 5 summarizes the results, with the top-performing outcomes in each dataset highlighted in bold. Among the baseline models, KNN and SVM exhibited poor classification performance for PNAR task. In contrast, CNN, LSTM, and their combinations demonstrated better classification accuracy. Both transformer-based models and the proposed method achieved excellent recognition results, particularly for dynamic activities like walking, running, and cycling, owing to the temporal stream’s capability to capture dependencies over a long period.

**Table 5:** Comparison with baseline methods

Models	WISDM		UCI-HAR		PAMAP2		KU-HAR		Average	
	Accuracy (%)	F1 (%)	Accuracy (%)	F1 (%)	Accuracy (%)	F1 (%)	Accuracy (%)	F1 (%)	Accuracy (%)	F1 (%)
KNN	82.88	82.65	84.24	84.12	83.21	82.87	81.24	81.76	82.89	82.85
SVM	83.23	83.34	85.26	85.38	84.37	84.42	82.22	82.51	83.77	83.91
Stack-HAR^16^	89.33	88.87	90.12	90.35	88.97	88.77	88.34	88.31	89.23	89.45
SDAE+LightGBM^47^	95.41	95.33	95.38	95.57	93.45	93.41	91.23	92.21	93.87	94.13
CNN^48^	95.13	95.21	95.87	95.99	94.89	94.94	92.34	92.89	94.56	94.76
LSTM^49^	95.78	96.12	96.83	96.89	95.41	95.72	94.76	94.89	95.69	95.90
CNNLSTM^50^	96.98	97.31	97.46	96.41	96.32	96.54	95.11	95.22	96.47	96.37
CNNLSTM + Attention^27^	97.41	98.28	98.21	98.47	96.66	96.87	95.88	95.92	97.04	97.39
Transformer^31^	98.17	98.21	98.54	98.52	98.01	98.17	97.87	97.92	98.14	98.20
CSSHAR^51^	98.22	98.29	98.87	98.92	98.41	98.57	98.03	98.18	98.38	98.49
Proposed	**99.32**	**99.12**	**99.51**	**99.21**	**98.87**	**98.77**	**98.62**	**98.26**	**99.08**	**99.22**

Bold shows the performance of the proposed method on different datasets.

The proposed method, utilizing a dual-stream transformer structure (comprising a time stream and a spatial stream), demonstrated a 1% accuracy improvement compared to the single-stream transformer structure (time stream alone). These findings highlight the proposed method’s strength and efficiency in learning both spatial and temporal stream features, enabling superior generalization across diverse datasets. The consistent improvements over traditional methods, including KNN, SVM, and hybrid deep learning models, validate the proposed method’s advantages.

## Discussion

With breakthroughs in technologies such as flexible electronics and biosensors, PNAR is expected to intersect with multiple disciplines such as genomics and metabolomics to promote the further development of precision medicine. This study introduces an innovative PNAR method that merges a TSCT with self-supervised contrastive pretraining to address the challenges of limited robustness and generalization posed by complex and variable activities. The proposed method effectively captures both spatial and temporal dependencies, enabling it to handle diverse and complex activity patterns across multiple datasets. The integration of self-supervised contrastive pretraining further reduced the reliance on large labeled datasets, enabling the model to effectively utilize unlabeled data and improve transferability across domains.

Extensive experiments on multiple heterogeneous datasets, such as WISDM, UCI-HAR, PAMAP2, and KU-HAR, confirmed the method’s superiority in accuracy, precision, recall, and F1 scores. Furthermore, cross-dataset evaluations demonstrated its exceptional generalization capability, demonstrating it suitability for diverse and significantly varying data distributions.

In the future, the proposed method can be enhanced by incorporating domain adaptation techniques to address more significant domain shifts and multi-task learning approaches to improve robustness. Furthermore, investigating the model’s scalability for real-time deployment and its performance in multi-sensor fusion scenarios would expand its practical applicability.

### Limitations of the study

Although the proposed TSCT combined with self-supervised contrastive learning exhibits good PNAR performance, there are still several limitations that need to be addressed in the future. This paper performs performance evaluation on multiple public datasets. Although these datasets cover a variety of activity types, their environments and application scenarios may differ from actual complex environments. Although the method proposed in the paper performs well in terms of accuracy, the model introduces relatively high computational complexity and model scale. Although the cost of online inference is acceptable for smartphones, the model may still face deployment challenges on ultra-low-power wearable devices or edge devices with strict memory and energy consumption constraints. The model’s computational efficiency needs to be further optimized to reduce the model’s inference time and storage requirements to ensure its feasibility in practical applications.

## Resource availability

### Lead contact

Further information and requests for resources, data, and code should be directed to and will be fulfilled by the lead contact, Jianquan Wang (wangjianquan@ustb.edu.cn).

### Materials availability

This study did not generate new materials.

### Data and code availability

• All data generated or analyzed during this study are included in this published article.
• This study does not report original code.
• Any additional information required to reanalyze the data reported in this study is available from the lead contact upon request.

## Acknowledgments

This work was supported in part by Science and Technology Innovation Program of Xiongan New Area (2025XAGG0028), 10.13039/501100001809National Natural Science Foundation of China (42401521, U25A20433, 92567203), Joint Research Fund for Beijing Natural Science Foundation and Haidian Original Innovation (L232001), Henan Key Research and Development Program (241111320700), 10.13039/501100021171Guangdong Basic and Applied Basic Research Foundation (2024A1515011866, 2024A1515011480, and 2025A1515011300), Central Guidance on Local Science and 10.13039/100006180Technology Development Fund of ShanXi Province (YDZJSX20231D005, and YDZJSX20231B017), S&T Program of Hebei (246Z0303G).

## Author contributions

Conceptualization, Q.W., J.W., and Z.J.; methodology, Q.W., J.M., and Y.B.B.; software, M.F. and Y.B.B.; investigation, J.M., Z.J., and H.L.; writing – original draft, Q.W., J.M., and Y.B.B.; writing – review and editing, all authors; visualization, M.F.; supervision, J.W., and H.L.; project administration, J.W. and M.F.; funding acquisition, Z.J. and H.L. All authors have read and agreed to the published version of the manuscript.

## Declaration of interests

The authors declare no competing interests.

## Declaration of generative AI and AI-assisted technologies in the writing process

The authors acknowledge the use of ChatGPT and Grammarly for language editing. After using this tool, the authors reviewed and edited the content as needed and take full responsibility for the content of the publication.

## STAR★Methods

### Key resources table

**Table:** 

REAGENT or RESOURCE	SOURCE	IDENTIFIER
**Software and algorithms**		

Python 3.12.3	Python Software Foundation	https://www.python.org
BYOL	Contrastive frameworks	https://github.com/google-deepmind/deepmind-research/tree/master/byol

### Method details

#### Problem modeling

Smartphones equipped with multiple sensors gather data from various sources. Thus, a sample *x* is represented as *x* = {*x*^(1)^,*x*^(2)^, …,*x*^(*n*)^}, where *n* denotes the complete count of directions across all sensors. For instance, if an accelerometer and a gyroscope are used, *n* = 6. Each *x*(*i*) corresponds to the data collected by a sensor in a specific direction. Since sensors typically sample at equal intervals, *x*(*i*) is further represented as *x*^(*i*)^ = {*x*^(*i*,1)^,*x*^(*i*,2)^, …,*x*^(*i*,*l*)^}. The data collected in a single direction from one sensor forms a one-dimensional time series of length *l*. Consequently, the overall sample is a multi-dimensional time series with a length of *l* and a dimension *n*, denoted as $x\in R^{l\times n}$. PNAR is a typical classification problem. We assume that the activity sample space is X, and the activity category space is *Y*, with |*Y*| representing the number of activity categories. Thus, an activity sample x∈X corresponds to an activity category y∈Y. The training set is represented as $D_{trian}=\left\{\left(x_{1},y_{1}\right),\left(x_{2},y_{2}\right),\ldots ,\left(x_{n},y_{n}\right)\right\}$. The aim is to establish a mapping function *f*:*X*→*Y* using $D_{trian}$, so that *f* aligns as consistent as possible with the real mapping relationship *f*_*real*_, namely *f* ˜ *f*_*real*_.

#### System architecture

**Figure 3:**
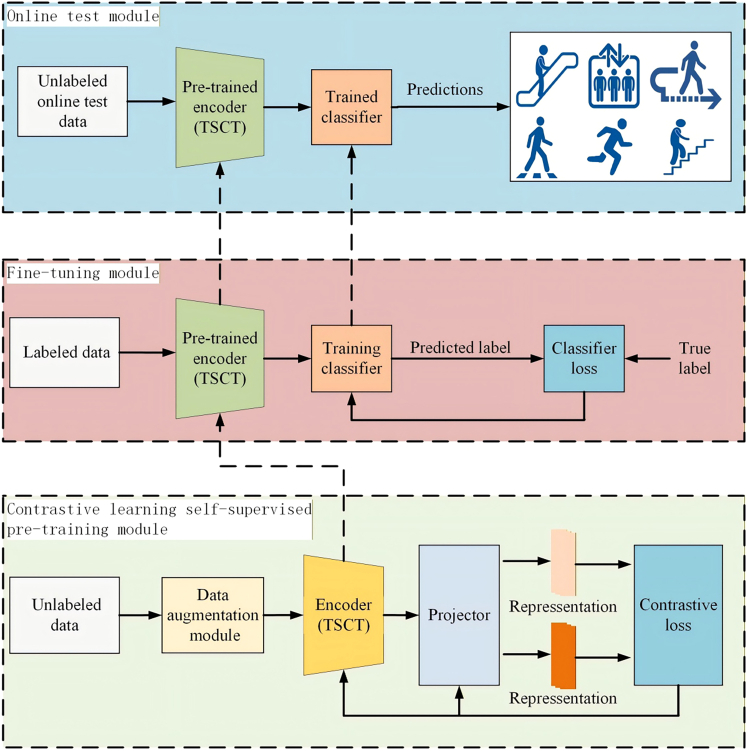
Proposed PNAR method based on two-stream transformer and contrastive learning

We present a PNAR method, depicted in Figure 3, is based on two-stream transformer and contrastive learning, which consists of three key modules: pre-training module, fine-tuning module, and online testing module, arranged sequentially from bottom to top. The pre-training module employs contrastive learning to train a deep feature encoder and mapper, transforming sensor signals into latent space representations. The module minimizes contrastive loss to optimize the latent space representation, clustering similar category features and dispersing different ones. Through this process, the encoder can effectively capture critical features from the input data, which are well-suited for downstream tasks. Finally, the online testing module applies the frozen encoder and the trained classifier from the fine-tuning stage to classify unlabeled sensor window signals, effectively identifying the corresponding activity categories.

#### Data pre-processing and augmentation

Data collected by sensors often contains data noise from hardware devices and unrelated activities. In addition, subtle vibrations produced involuntarily during activities may also affect the accuracy of the network model. Therefore, we use fourth-order Butterworth bandpass filtering to remove attenuated data Direct Current (DC) drift, data artifacts due to motion, and other low- and high-frequency noise.

Sampling rates vary between different sensors and different datasets. To unify these signals into a multidimensional matrix as input to the model, we convert all signals to a consistent sampling frequency by upsampling or downsampling. This article unifies the sampling rates of different sensors and datasets to 100Hz. In addition, due to differences in units and dimensions between different sensing data, we perform maximum-minimum normalization on the data.

Sliding window is a commonly used data segmentation method. The window’s size and overlap significantly impact mode’s predictive performance. This paper uses multi-scale sliding windows for data segmentation, in which samples are obtained by sliding under the time dimension of small-scale windows, while retaining the characteristics of large-scale time windows that maintain non-repetitive motion periodic activities, thereby obtaining richer feature.

In the temporal stream, since the self-attention mechanism has difficulty exploiting the sequential correlations of time steps, some temporally sensitive actions are difficult to recognize. Positional Encoding (PE) is added to the nonlinear transformation of the temporal stream sequence data to focus on the temporal sequence information of the data. Since the signals from each sensor axis contribute differently to the recognition results, we add sensor attention to the spatial stream sequence data to focus on the contribution of each sensor axis within the input time window.

#### Two-stream convolutional transformer model

**Figure 4:**
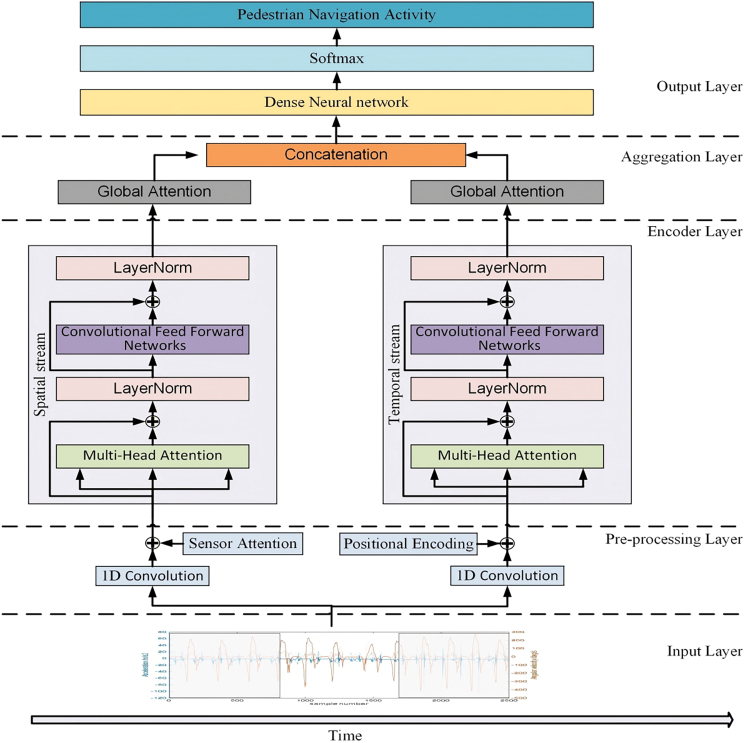
Proposed TSCT model

To derive implicit high-level features, we propose a Two-Stream Convolutional Transformer (TSCT) model, as depicted in Figure 4. The spatial stream captures multi-modal sensor dependencies, while the temporal stream leverages attention mechanism to excavate temporal relationships. The TSCT model consists of five layers: input, preprocessing, encoder, aggregation, and prediction layer. When employed as an encoder in a contrastive self-supervised learning module, the TSCT model includes only the first four layers, excluding the prediction layer. The input layer processes sensor window data independently and feeds it into the dual-stream channels. The Transformer encoder layer extracts distinct features from each stream, which are then integrated in the aggregation layer using global attention mechanisms. Finally, the prediction layer produces the final classification results. The following sections will offer a d etailed explanation of each layer.

##### Transformer encoder layer

The Transformer encoder layer includes a multi-head attention module with a residual structure and a convolutional feedforward module. The Transformer encoder layer utilizes attention mechanism to capture global information from sensor data. The multi-head attention module calculates the similarities between time steps and allocates weights according to the sequence’s internal correlations. This layer computes dependencies of all time steps within a time window, and captures the overall sensor information. First, the time series features generated by preprocessing layer are linearly transformed to produce three matrices: ***Q*** as the query matrix, ***K*** as key matrix, and ***V*** as value matrix:

(Equation 1) $$Q=XW^{Q},K=XW^{k},V=XW^{V}$$

where $W^{Q}\in R^{d\times d_{k}},W^{K}\in R^{d\times d_{k}}$ and $W^{V}\in R^{d\times d_{\nu }}$ represent weight matrices that can be learned in linear transformation, respectively. where X is the layer input (dimension $R^{b_{s}*s_{d}*d_{m}}$ for spatial stream, $R^{b_{s}*w_{l}*d_{m}}$ for temporal stream). Vector similarity is calculated by taking the dot product of Q and K. Subsequently, scale *QK*^⊤^and SoftMax function are applied to generate attention scores for the corresponding time step. Finally, the attention score is multiplied by the value (***V***) for weighting operation. The overall calculation process is as follows:

(Equation 2) $$\text{Attention}\left(Q,K,V\right)=\text{softmax}\left(\frac{QK^{T}}{\sqrt{d_{k}}}\right)V$$

where *d*_*k*_ denotes the size of query matrix ***Q*** and key matrix ***K***. *d*_*ν*_ denotes the size of value matrix ***V***. The input of the temporal stream channel is denoted as X, while the input of the spatial stream channel is denoted as Y.

To enhance the model’s capacity to concentrate on diverse areas, we execute *h* attention modules in parallel. We use different weight matrices when performing linear transformations to produce multiple ***Q***, ***K***, and ***V***. Each attention module computes its output as ***Z***_*i*_ = Attention⁡(***Q***_*i*_,***K***_*i*_,***V***_*i*_). Finally, the multi-head attention module concatenates the outputs from each attention head and applies another linear transformation as follow:

(Equation 3) $$f_{mha}=Multi-head\;Attention\left(Q,K,V\right)=W_{o}*Contact\left(Z_{1},Z_{2},\cdots ,Z_{h}\right)$$

where $W_{o}\in R^{hd_{v}\times d_{mut}}$ is the corresponding projection matrix. To mitigate the vanishing gradient problem caused by module stacking and preserve information integrity, the attention module incorporates residual connections, ensuring that *d* = *d*_*out*_.

We combine the attention module’s output with the input sequence and perform layer normalization (LN) operation before passing it to the feedforward layer containing residual connections. The result is recorded as ***O***_*a*_:

(Equation 4) $$O_{a}=\mathrm{LN}\left(X+O_{h}\right)$$

In the standard Transformer architecture, the feedforward layer includes two fully connected layers. To enhance local information extraction, we introduce a convolutional feedforward network (CFN) module. CFN consists of two one-dimensional convolutional layers, each equipped with batch normalization (BN), ReLU activation, and dropout to mitigate overfitting. Its output is recorded as ***O***_*f*_. The complete CFN computation is described as follows:

(Equation 5) $$\left\{ \begin{array}{c} O=\text{ReLU}\left(\text{BN}\left(\text{Conv}\left(W_{1};O_{a}\right)\right)\right)\\ O_{f}=\text{Dropout}(\text{ReLU}\left(\text{BN}\left(\text{Conv}\left(W_{2};O\right)\right)\right) \end{array}\right.$$

where ***W***_1_ and ***W***_2_ are learnable weight that are convolved twice. We add CFN module’s output ***O***_*f*_to input sequence ***O***_*a*_ and perform layer normalization to obtain the final features of the Transformer encoder layer, denoted as ***X***_*e*_:

(Equation 6) $$X_{e}=\mathrm{LN}\left(O_{a}+O_{f}\right)$$

Due to the use of residual connections in the Transformer encoder layer, the dimensions of the extracted features remain unchanged.

##### Aggregation layer

Aggregation layer processes features extracted from the spatial stream channel (with dimensions $R^{b_{s}*s_{d}*d_{m}}$) and temporal stream channel (with dimensions $R^{b_{s}*w_{l}*d_{m}}$). To facilitate effective feature fusion, a global attention mechanism is utilized to enhance relevant information in the extracted features while suppressing irrelevant details. First, linear projection is performed on ***X***_*e*_ to calculate position-specific weights. Then These weights are normalized using the SoftMax function. Finally, we calculate the weighted feature matrix C through the Hadamard product as follows:

(Equation 7) $$C=\text{softmax}\left(X_{e}W_{3}^{T}+b_{3}\right)⊙X_{e}$$

The attention output is produced by multiplying the feature weight matrices with the respective hidden vectors.

(Equation 8) $$F=\sum_{i=1}^{h}Z_{i}\cdot C_{i}$$

To leverage the complementarity of temporal and spatial information, we concatenate spatial and temporal features into a unified feature vector as the output of aggregation layer U:

(Equation 9) $$U=contact\left[F_{S};F_{T}\right]$$

where *F*_*S*_ represents the spatial stream feature vector, while *F*_*T*_ denotes the temporal stream feature vector.

##### Output layer

This study constructs the TSCT as a complete model that allows recognition results to be obtained directly from sensor data. Because PNAR is a typical multi-class classification task, its prediction layer is a fully connected layer with SoftMax unit. The fully connected layer adjusts the classification result, allowing SoftMax units to predict the activity categories. We use the standard cross entropy loss function to calculate the distance L between predicted value and actual value *y*_*i*_:

(Equation 10) $$L=-\sum_{i}^{|L|}y_{i}\;\log\left(f\left(\theta ;X_{T};X_{S}\right)\right)$$

where ∣*L*∣ represents the overall count of categories. *f*(·) represents the distribution of prediction. *X*_*T*_ is the inputs of temporal stream and *X*_*S*_ is the inputs of spatial stream.

#### Self-supervised contrastive pretraining method

Pretraining is crucial for improving model generalization, especially when labeled data is limited or when there are significant differences between datasets. MoCo^32^ reformulated several prior contrastive learning methods as a dictionary lookup problem. By utilizing a queue and treating multiple elements within it as negative samples, this approach mitigates computational overhead. BYOL^35^ directly removes negative samples from MoCo, setting up two distinct networks: an online network and a target network. After obtaining features through the projection head, the online network adds an additional prediction layer composed of one or two fully connected layers. This prediction layer is then used to predict the features obtained by the target network, effectively performing a regression task. The model employs MSE as its loss function. Therefore, we design a self-supervised contrastive pretraining method under BYOL framework that leverages unlabeled data to develop a robust feature encoder, which effectively captures the inherent similarities and differences.

##### Contrastive loss function

The model employs a normalized cross-entropy loss function to optimize encoder. For each batch of samples ${\left\{x_{i}\right\}}_{i=1}^{N}$, the loss is calculated as follows:

(Equation 11) $$L_{i,j}=-\log\frac{\exp\left(\text{sim}\left(z_{i},z_{j}\right)/\tau \right)}{\sum_{k=1}^{2N}1_{\left[k\neq i\right]}\;\exp\left(\text{sim}\left(z_{i},z_{k}\right)/\tau \right)}\text{,}$$

where *z*_*i*_ and *z*_*j*_ are the latent representations of the augmented input samples, *sim*(·,·) denotes cosine similarity, and *τ* is a temperature parameter.

##### Encoder design

The TSCT model serves as the encoder in the pretraining stage, leveraging its spatial and temporal streams to extract comprehensive features. Encoders effectively mine local and global features from input signals through self-attention mechanisms.

#### Supervised fine-tuning

After pre-training, the encoder containing the TSCT architecture is fine-tuned under supervised conditions to adapt to specific PNAR tasks. This phase ensures that the pretrained features are aligned with the target dataset’s label distribution and optimized for classification. A simple fully connected layer, often referred to as a linear classifier, is appended to the output of the pretrained encoder. This classifier projects the latent feature representations into the label space corresponding to the activity classes. For datasets with *C* activity classes, the output layer has *C* units, followed by a softmax activation function for probability estimation.

(Equation 12) $$\hat{y}=\text{softmax}\left(W\cdot z+b\right)$$

where *z* represents the latent representation derived from the pretrained encoder. W and b are weight matrix and bias vector, respectively. The fine-tuning phase optimizes the cross-entropy loss function:

(Equation 13) $$L_{\text{CE}}=-\frac{1}{N}\sum_{i=1}^{N}\sum_{c=1}^{C}y_{i,c}\;\log\left({\hat{y}}_{i,c}\right)\text{,}$$

where *y*_*i*,*c*_ represents the actual label encoded in one-hot format and ${\hat{y}}_{i,c}$ denotes the estimated likelihood for class *c*.

### Quantification and statistical analysis

We completed quantitative analysis based on Python (PyTorch framework), and conducted model training and evaluation on workstations equipped with NVIDIA GPUs. We use Accuracy, Precision, Recall and F1-score as the main evaluation indicators. To avoid accidental results, we use 5 repetitive Leave-One-Subject-Out Cross-Validation (LOSO-CV) tests to evaluate the performance of the proposed method, and the final results are presented as “mean ± standard deviation”. We use confusion matrix to visually analyze the recognition accuracy on different datasets. In the cross-dataset experiment, after the model was trained on one dataset, it was tested on another dataset without additional domain adaptation to verify the generalization ability of the proposed method under different sensor configurations and activity distribution conditions. To evaluate the performance of the proposed method under different scales of labeled data, the proposed model was trained using 2%–20% of labeled data, respectively. Table 4 summarizes the accuracy variations on the WISDM, UCI-HAR, PAMAP2, and KU-HAR datasets.
